# A role for immunotherapy: preventing macrophage anergy in the tumour environment

**DOI:** 10.1186/2051-1426-1-S1-P185

**Published:** 2013-11-07

**Authors:** Androulla Elia, James Mapes, Thomas Knorpp, Manfred Schmolz, Eric Garcia, Thorsten Hagemann, Laura Rosa Brunet

**Affiliations:** 1 St George's, University of London, London, UK; 2 Myriad RBM, Austin, TX, USA; 3 EDI GmbH, Reutlingen, Germany; 4 BioElpida, Dardilly, France; 5 Barts Cancer Institute, QMUL, London, UK; 6 Immodulon Therapeutics Ltd, London, UK

## 

The tumour microenvironment is shaped by the interaction of tumour cells with a variety of “non” malignant cellular and acellular components. It is the secretion of cytokines, chemokines, metabolites and other mediators in the tumour stroma that shapes the natural evolution of cancer. Whereas the tumour strives to promote an immunosuppressive environment to limit anti-tumour immune response, infiltrating immune cells aim to control tumour growth. The balance between these two conflicting interests shapes the immune contexture of cancer and ultimately patient prognosis. In an effort to characterize the role played by macrophages, we have developed an in vitro system using human pancreatic tumour cell lines and a human monocyte-macrophage leukemic cell line (ThP1). We have investigated the immunosuppressive properties of pancreatic cancer cell lines and how the immune properties of macrophages are influenced by the tumour supernatant in vitro. We have applied our findings to a clinically relevant murine model of pancreatic cancer. We found that, despite some heterogeneity, the profile of MIAPACA-2, BxPC3 and PANC-1, included IL-8, MCP-1, SCF and VEGF secretion. Not surprisingly, when ThP1 cell were cultured in the presence of increasing concentrations of tumour supernatant and stimulated with LPS, we observed a significant downregulation of their ability to produce cytokines associated with an M1 profile (e.g. IL-6, IL-12p70, IL-23, TNF-α). However, ThP1 also failed to display an M2 profile; IL-10 and IL-1ra were similarly downregulated. ThP1 stimulated with LPS in the presence of tumour supernatant also had impaired secretion of a number of other cytokines such as IL-18, MIP-1α and MIP-1β and MCP-1. In contrast, they appeared to maintain secretion of VEGF and IL-7. Next we investigated how the immune stimulant IMM-101, an immunotherapeutic agent comprising heat-killed whole cell Mycobacterium obuense, might be able to shift this anergic phenotype. IMM-101 is currently under clinical evaluation in two Phase II clinical trials in pancreatic (EudraCT number 2010-022757-42) and colorectal cancer (EudraCT number 2011-003958-85). We found that despite the presence of tumour supernatant, macrophages stimulated with IMM-101 retained the ability to secrete the full range of cytokines and we did not observe the impairment reported after LPS stimulation. We believe the immunological properties of IMM-101 make this a potential candidate for successful immunotherapy.

